# HLA-DR Presentation of the Tumor Antigen MSLN Associates with Clinical Outcome of Ovarian Cancer Patients

**DOI:** 10.3390/cancers14092260

**Published:** 2022-04-30

**Authors:** Christian M. Tegeler, Jonas Scheid, Hans-Georg Rammensee, Helmut R. Salih, Juliane S. Walz, Jonas S. Heitmann, Annika Nelde

**Affiliations:** 1 Clinical Collaboration Unit Translational Immunology, German Cancer Consortium (DKTK), Department of Internal Medicine, University Hospital Tübingen, 72076 Tübingen, Germany; christian.tegeler@med.uni-tuebingen.de (C.M.T.); helmut.salih@med.uni-tuebingen.de (H.R.S.); juliane.walz@med.uni-tuebingen.de (J.S.W.); 2 Department of Obstetrics and Gynecology, University Hospital Tübingen, 72076 Tübingen, Germany; 3 Department of Peptide-Based Immunotherapy, University Hospital Tübingen, 72076 Tübingen, Germany; jonas.scheid@student.uni-tuebingen.de (J.S.); annika.nelde@uni-tuebingen.de (A.N.); 4 Institute for Cell Biology, Department of Immunology, University of Tübingen, 72076 Tübingen, Germany; hans-georg.rammensee@uni-tuebingen.de; 5 Quantitative Biology Center (QBiC), University of Tübingen, 72076 Tübingen, Germany; 6 Cluster of Excellence iFIT (EXC2180) “Image-Guided and Functionally Instructed Tumor Therapies”, University of Tübingen, 72076 Tübingen, Germany; 7 German Cancer Consortium (DKTK) and German Cancer Research Center (DKFZ), Partner Site Tübingen, 72076 Tübingen, Germany

**Keywords:** ovarian cancer, mucin-16, mesothelin, immunopeptidome, survival, clinical data

## Abstract

**Simple Summary:**

The immunopeptidome represents the entirety of peptides that are presented on the surface of cells on human leukocyte antigen (HLA) molecules and are recognized by the T-cell receptors of CD4^+^ and CD8^+^ T-cells. Malignant cells present tumor-associated antigens essential for tumor immune surveillance, which can be targeted by T-cell-based immunotherapy approaches. For ovarian carcinomas, various tumor-associated antigens, such as Mucin-16 and Mesothelin, have been described. The aim of our study is to analyze immunopeptidome-defined tumor antigen presentation in ovarian carcinoma patients in relation to clinical characteristics and disease outcomes to define potential biomarkers. Our work demonstrates the central role of HLA-DR-restricted peptide presentation of the tumor antigen Mesothelin and of CD4^+^ T-cell responses for tumor immune surveillance, and underlines Mesothelin as a prime target antigen for novel immunotherapeutic approaches for ovarian carcinoma patients.

**Abstract:**

T-cell recognition of HLA-presented antigens is central for the immunological surveillance of malignant disease and key for the development of novel T-cell-based immunotherapy approaches. In recent years, large-scale immunopeptidome studies identified naturally presented tumor-associated antigens for several malignancies. Regarding ovarian carcinoma (OvCa), Mucin-16 (MUC16) and Mesothelin (MSLN) were recently described as the top HLA class I- and HLA class II-presented tumor antigens, respectively. Here, we investigate the role and impact of immunopeptidome-presented tumor antigens on the clinical outcomes of 39 OvCa patients with a follow-up time of up to 50 months after surgery. Patients with a HLA-restricted presentation of high numbers of different MSLN-derived peptides on their tumors exhibited significantly prolonged progression-free (PFS) and overall survival (OS), whereas the presentation of MUC16-derived HLA class I-restricted peptides had no impact. Furthermore, a high HLA-DRB gene expression was associated with increased PFS and OS. In line, in silico prediction revealed that MSLN-derived HLA class II-presented peptides are predominantly presented on HLA-DR allotypes. In conclusion, the correlation of MSLN tumor antigen presentation and HLA-DRB gene expression with prolonged survival indicates a central role of CD4^+^ T-cell responses for tumor immune surveillance in OvCa, and highlights the importance of immunopeptidome-guided tumor antigen discovery.

## 1. Introduction

The breakthrough clinical success of T-cell-based immunotherapy approaches, i.e., immune checkpoint inhibitors, bispecific antibodies and adoptive T-cell transfer, has caused a profound change in oncological treatment [1,2,3,4,5]. These therapies rely on the rejection of cancer cells through the recognition of tumor antigens and T-cell-mediated cytotoxicity. Tumor antigens are represented by human leukocyte antigen (HLA)-independent surface molecules or by HLA class I- and HLA class II-presented T-cell epitopes, originating from intracellular proteins [6]. In terms of the latter, neoepitopes derived from tumor-specific nonsynonymous somatic mutations are considered as central specificities of T-cell responses that are therapeutically reinforced by checkpoint inhibition, the only one of the above-described T-cell-mobilizing strategies that is thoroughly effective in solid tumors to date [7,8]. As only a minority of mutations at the DNA level is translated and naturally processed to HLA-presented neoepitopes targetable for T-cells [9,10], non-mutated tumor-associated antigens, which result from differential gene expression or protein processing in tumor cells, are receiving increasing attention. In recent years, the identification of naturally presented tumor antigens has been advanced by mass spectrometry-based analyses of the entirety of HLA class I- and HLA class II-presented peptides, termed the immunopeptidome [11,12,13,14,15,16,17]. This methodology enabled the identification of highly frequent tumor-associated antigens for various tumor entities [11,12,13,14,15,16,17], which have been used for the development of novel T-cell-based immunotherapy approaches [18,19]. Several studies demonstrate the pathophysiological relevance of T-cells targeting such tumor antigens by correlating spontaneous, pre-existing and immune checkpoint inhibitor-induced antigen-specific T-cell responses with improved clinical outcomes [11,20,21,22]. However, the role of tumor antigen presentation in the immunopeptidome on patient survival and outcomes has not yet been investigated. Here, we report on the clinical characteristics and disease outcomes of 39 ovarian carcinoma (OvCa) patients in relation to immunopeptidome-defined tumor antigen presentation. In a recent study, the mass spectrometry-based characterization of the antigenic landscape revealed several OvCa-associated CD4^+^ and CD8^+^ T-cell epitopes from a panel of tumor-associated antigens [16]. Thereby, Mesothelin (MSLN) and Mucin-16 (MUC16), also known as cancer antigen 125 (CA-125), were identified as the two top OvCa-associated antigens presented on approximately 80% and 50% of analyzed tumor samples, respectively [16]. In the present study, we show that the presentation of MSLN-derived HLA class II-restricted peptides in the immunopeptidome is associated with longer patient survival, providing important insights into the role of immunopeptidome-guided CD4^+^ T-cell responses for tumor immune surveillance in OvCa.

## 2. Materials and Methods

### 2.1. Clinical Data

The clinical and survival data of patients with OvCa, whose tumor tissues had been previously analyzed by immunopeptidomics and RNA sequencing analysis [16], were collected at the University Hospital Tübingen with a follow-up phase of up to 50 months after surgery. Informed consent was obtained in accordance with the Declaration of Helsinki protocol. The study was approved by and performed according to the guidelines of the local ethics committees (353/2007/B02). Detailed donor characteristics are provided in Table 1.

### 2.2. Immunopeptidome and RNA Sequencing Data

Immunopeptidome and RNA sequencing data were retrieved from a previous publication [16]. The immunopeptidome dataset comprised the total number of identified OvCa-associated HLA class I- and HLA class II-presented peptides per patient, as well as the number of HLA class I- and HLA class II-presented MUC16- and MSLN-derived peptides per patient, representing the two top OvCa-associated tumor antigens. The OvCa-associated antigens include the previously described 56 and 28 HLA class I- and HLA class II-presented antigens identified exclusively and high frequently in OvCa, respectively (Table S1). Progression-free (PFS) and overall survival (OS) was depicted for < and ≥ median of the number of presented peptides (immunopeptidome data) and for < and ≥ median of FPKM values (RNA sequencing data). Since variable amounts of tumor tissue were employed for the immunopeptidome analyses (range: 0.5 g–3.0 g, median: 1.8 g) and a positive correlation of sample mass and identified HLA-presented peptides were observed (Figure S1), the analyses were conducted with the number of peptides normalized to 1 g of tumor tissue.

### 2.3. Binding Prediction

HLA class II MSLN peptide-binding prediction was performed for all previously described MSLN-derived peptides (*n* = 62) [16] using the prediction algorithm NetMHCIIpan 4.1a [23] with all available allele combinations (*n* = 660 for HLA-DR, *n* = 2912 for HLA-DQ, *n* = 2048 for HLA-DP) and the threshold of percentile rank <5.

### 2.4. Software and Statistical Analysis

Data are displayed as the mean with standard deviation, box plots as median with 25th and 75th quantiles and min/max whiskers. Continuous data were tested for distributions and individual groups were tested by use of the Mann–Whitney U-test. PFS and OS were calculated by the Kaplan–Meier method. The log-rank test was performed to test the difference of survival between the groups. Graphs were plotted using GraphPad Prism v.9.1.2. Statistical analyses were conducted using JMP Pro (SAS Institute, v.14.2) software. Overlap analysis was performed using BioVenn [24]. *p*-values ≤ 0.05 were considered statistically significant.

## 3. Results

### 3.1. Characteristics of the Patient Cohort

We analyzed the clinical and survival data of 39 patients with OvCa (Table 1) in relation to tumor antigen presentation within the HLA class I and HLA class II immunopeptidome [16]. All patients underwent surgery at primary diagnosis between 2010 and 2015 and received a systemic therapy with platinum-based chemotherapy. The median patient’s age on the day of surgery was 62 years. Most patients were post-menopausal (82%). The majority of patients had lymph node metastases (66%), whereas distant metastases were detected in only 21% of patients. Patients were followed up for up to 50 months after surgery. The median PFS was 293 days and the median OS was 921 days with a maximal follow-up time of 1500 days.

### 3.2. Tumor Antigen Presentation Does Not Associate with Demographics and Tumor Characteristics of OvCa

The association of demographics and tumor characteristics of OvCa patients with tumor antigen presentation was analyzed using sample mass-normalized mass spectrometry-derived immunopeptidome data of total OvCa-associated HLA class I- and HLA II-restricted peptides, as well as MUC16-derived HLA class I- and MSLN-derived HLA class II-restricted peptides. OvCa-associated peptides describe the 56 and 28 HLA class I- and HLA class II-restricted antigens previously described to be exclusively and highly frequently presented in OvCa [16].

**Figure 1:**
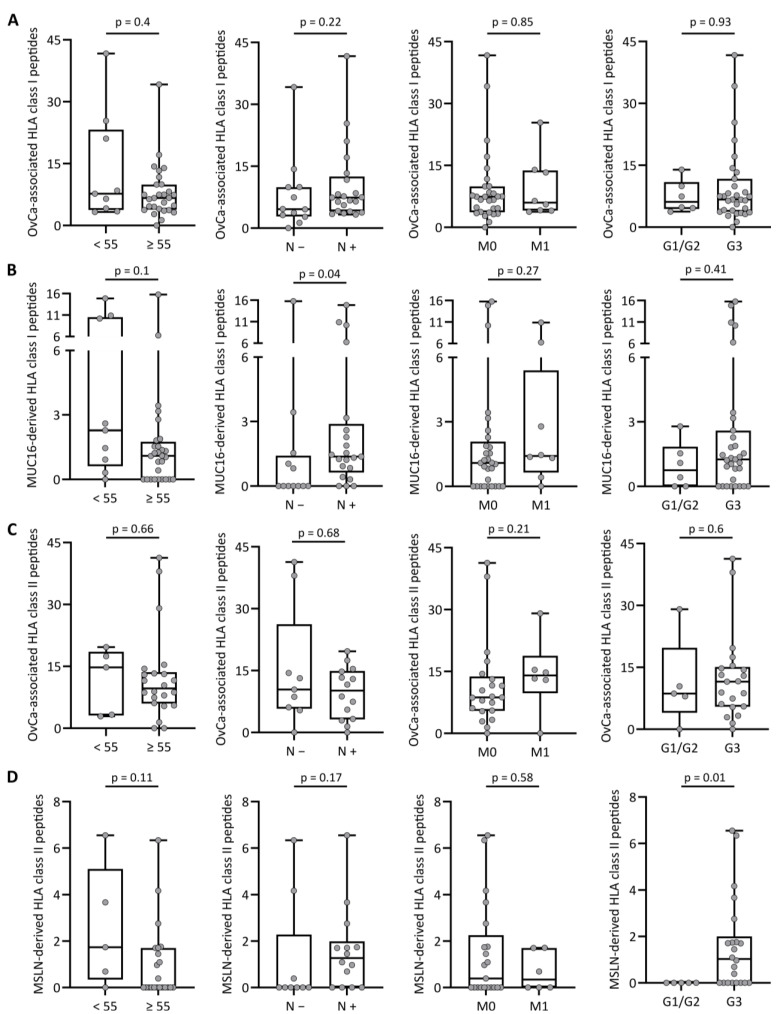
HLA presentation of tumor-associated antigenic peptides according to demographics and tumor characteristics. Number of (**A**) OvCa-associated and (**B**) MUC16-derived HLA class I-presented peptides, as well as (**C**) OvCa-associated and (**D**) MSLN-derived HLA class II-presented peptides identified by the immunopeptidome analysis of primary tumor tissue in relation to demographics and tumor characteristics, including age (left panel), nodal metastases (mid-left panel), distant metastases (mid-right panel) and tumor grading (right panel). Dots represent data from individual patients. Boxes represent the median and 25th to 75th percentiles, whiskers are minimum to maximum, Mann–Whitney U-test. Abbreviations: <55, age below 55 years; ≥55, age equal to or over 55 years; N −, no lymph node metastases; N +, lymph node metastases; M0, no sign of distant metastases; M1, distant metastases; G1/2, low/intermediate tumor grading; G3, high tumor grading; and *p*, *p*-value.

The presentation of total OvCa-associated HLA class I-restricted peptides did not differ according to the demographics and tumor characteristics, including age, nodal status, distant metastasis and grading (Figure 1A), as well as the location of primary disease, menopausal status and familial predisposition (Figure S2A). Regarding MUC16-derived HLA class I peptides, we found that cancers of nodal-positive patients presented more MUC16-derived peptides than those of patients without lymph node metastases (*p*-value 0.04, Figure 1B), whereas no differences were found for the other parameters (Figure 1B and Figure S2B).

In line with HLA class I data, the presentation of OvCa-associated HLA class II-restricted peptides was not associated with demographics and tumor characteristics (Figure 1C and S2C). However, for MSLN-derived HLA class II-presented peptides, we observed that cancers of patients with high tumor grading (G3) present significantly more MSLN-derived HLA class II peptides than those of patients with low or intermediate tumor grading (G1/G2) (*p*-value: 0.01, Figure 1D). No difference in MSLN-derived HLA class II peptide presentation could be observed for age, nodal positivity and distant metastasis (Figure 1D), as well as for the location of primary disease, menopausal status or familial predisposition (Figure S2D). Taken together, the MUC16 and MSLN tumor antigen presentations within the HLA class I and HLA class II immunopeptidomes of OvCa were not associated with demographics and the majority of tumor characteristics, except for nodal status and grading, respectively.

### 3.3. Presentation of MSLN-Derived HLA Class II-Restricted Peptides Is Associated with an Improved Patient Survival

**Figure 2:**
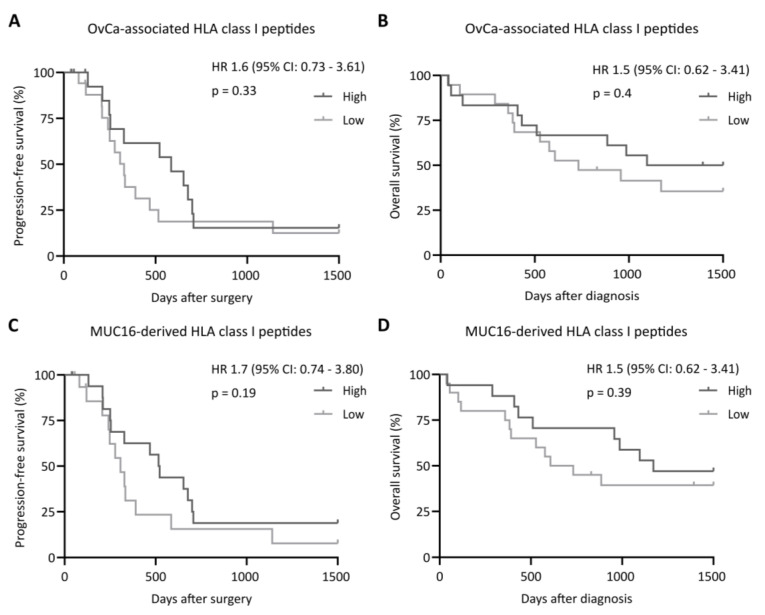
Impact of tumor-associated and MUC16-derived HLA class I-restricted peptide presentation on clinical outcomes. Impact of (**A**,**B**) OvCa-associated and (**C**,**D**) MUC16-derived HLA class I-restricted peptide presentation on (**A**,**C**) progression-free and (**B**,**D**) overall survival rates, according to antigenic peptide presentation. Kaplan–Meier analysis, log-rank test. Abbreviations: HR, hazard ratio; CI, confidence interval; and *p*, *p*-value.

**Figure 3:**
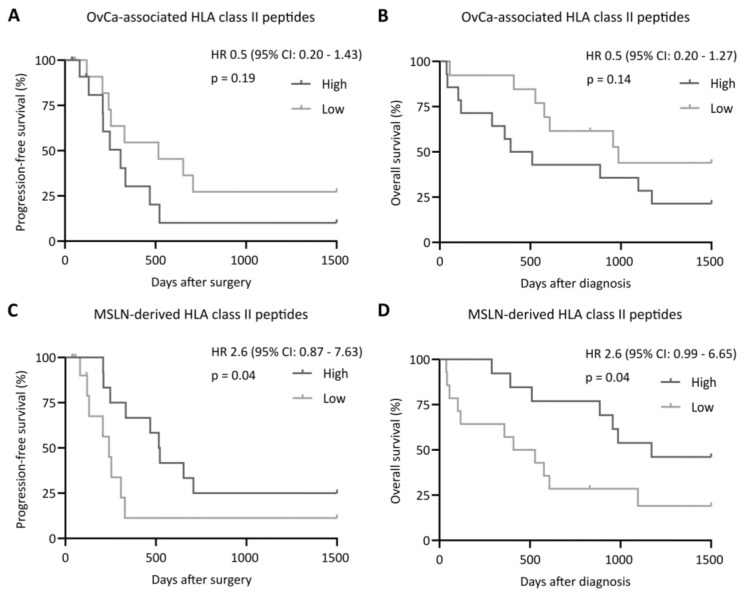
Impact of tumor-associated and MSLN-derived HLA class II-restricted peptide presentation on clinical outcomes. Impact of (**A**,**B**) OvCa-associated and (**C**,**D**) MSLN-derived HLA class II-restricted peptide presentation on (**A**,**C**) progression-free and (**B**,**D**) overall survival rates, according to antigenic peptide presentation. Median progression-free and overall survival rates in patients presenting high numbers of MSLN-derived peptides in their tumor immunopeptidomes were 519 and 1171 days after surgery or diagnosis, respectively. Kaplan–Meier analysis, log-rank test. Abbreviations: HR, hazard ratio; CI, confidence interval; and *p*, *p*-value.

Subsequently, we investigated the impact of tumor antigen-derived HLA-restricted peptide presentation on PFS and OS. For HLA class I, PFS and OS rates did not differ between the patient groups with a low or high immunopeptidome presentation of OvCa-associated (Figure 2A,B) or MUC16-derived peptides (Figure 2C,D). Similar observations were made for the total OvCa-associated HLA class II peptides, with no significant difference between a low or high tumor antigen presentation regarding PFS and OS (Figure 3A,B). For MSLN-derived HLA class II-restricted peptides, we observed a significantly improved PFS and OS in patients with tumors presenting higher numbers of these peptides (*p*-value: 0.04 and 0.04, respectively, Figure 3C,D), highlighting the importance of MSLN-directed CD4^+^ immune responses for tumor immunosurveillance.

### 3.4. HLA-DR Is a Predictor for Overall Survival in OvCa Based on Preferential HLA-DR-Restricted MSLN Presentation

**Figure 4:**
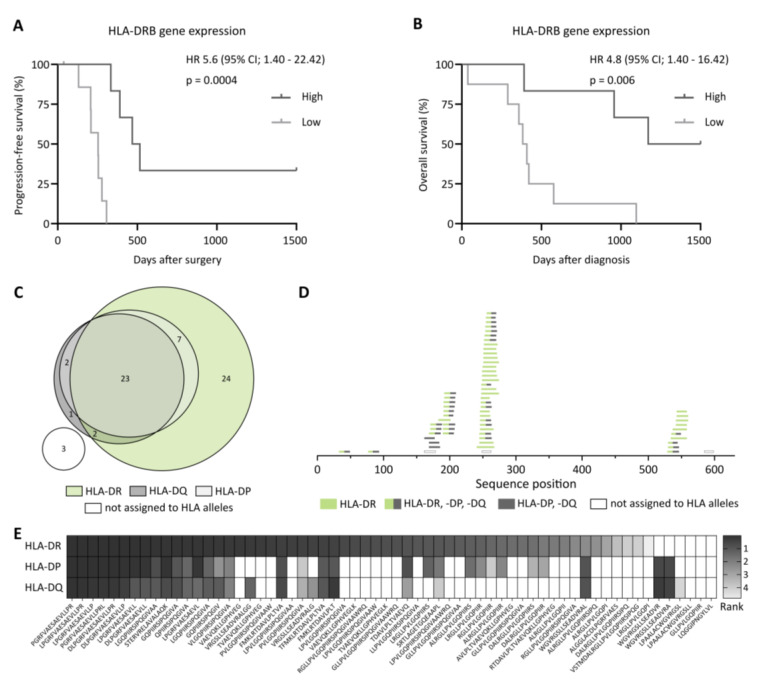
Impact of HLA-DRB gene expression on clinical outcome and in silico prediction of MSLN-derived peptide binding to HLA class II allotypes. (**A**,**B**) Impact of HLA-DRB gene expression level on (**A**) progression-free and (**B**) overall survival rates, according to low and high HLA-DRB gene expressions in the patients’ tumors. Median progression-free and overall survival in patients with a high HLA-DRB gene expression in their tumor was 493 and 1136 days after surgery or diagnosis, respectively. Kaplan–Meier analysis, log-rank test. (**C**) Overlap analysis of MSLN-derived peptides (*n* = 62) predicted in silico to bind to HLA-DR, -DP or -DQ alleles. (**D**) Peptide clustering of HLA-presented peptides on the MSLN source protein sequence according to in silico predicted binding to HLA class II allotypes. Peptides are grouped according to their predicted binding to different alleles of HLA-DR, -DP or -DQ. Peptides marked in green are predicted to bind exclusively to HLA-DR alleles. (**E**) Heat map depicting the percentile rank of the prediction scores for MSLN-derived peptides (*n* = 62) according to HLA-DR, -DP and -DQ allele groups. For each group, the rank of the best predicted allele is shown. Peptides are sorted by descending binding to HLA-DR alleles. Peptides with a percentile rank <1 are identified as strong binders, peptides with a percentile rank <5 as weak binders. The gradient ranges from strong HLA binding (dark gray) over weak HLA binding (light gray) to no HLA-restricted peptide binding (white). Abbreviations: HR, hazard ratio; CI, confidence interval; and *p*: *p*-value.

The analysis of complex immunopeptidomics data has not yet become part of routine clinical practice and therefore, to date, does not constitute a suitable biomarker. Therefore, we analyzed the RNA sequencing data of the tumor antigen MSLN in regard to the clinical outcomes. Of note, the level of MSLN gene expression, in contrast to HLA-restricted MSLN presentation, had no impact on patient survival (Figure S3), consistent with the previously reported distorted correlation of gene expression and HLA-restricted peptide presentation [25,26,27,28]. However, the expression level of HLA molecules themselves, which are crucial for tumor antigen-derived peptide presentation, might be a suitable predictor. For HLA class I genes (HLA-A, -B and -C, Figure S4) and the majority of HLA class II genes (HLA-DRA, -DPA, -DPB, -DQA and -DQB, Figure S5), a correlation of gene expression and patient survival was not detected. However, we observed improved PFS and OS for patients with a high HLA-DRB gene expression in their tumors (Figure 4A,B). To investigate if the 62 previously described HLA class II-presented MSLN-derived peptides are presented on HLA-DR allotypes, we performed an in silico binding prediction using the NetMHCIIpan 4.1a algorithm [23], considering 5620 different HLA class II alleles (n = 660 for HLA-DR, n = 2912 for HLA-DQ, *n* = 2048 for HLA-DP). A total of 95.2% (59/62) of the described peptides were predicted to bind to a total of 5,488 different HLA class II alleles. Interestingly, 94.9% (56/59) of those peptides were predicted to bind to different HLA-DR alleles, whereas only 54.2% (32/59) and 47.5% (28/59) were predicted to bind to HLA-DP and HLA-DQ alleles, respectively, highlighting the preferential HLA-DR-restricted presentation of MSLN-derived peptides (Figure 4C–E, Supplementary Data S1).

## 4. Discussion

T-cell recognition of HLA-presented antigens is central for the immunological surveillance of malignant disease [25,26]. Various immunotherapeutic approaches, i.e., adoptive T-cell transfer and vaccination approaches, intend to identify and target suitable tumor antigens to therapeutically induce an anti-tumor T-cell response [1,18,27,28]. The mass spectrometry-based characterization of naturally presented HLA-restricted peptides enabled the definition of tumor-associated antigens for several malignancies [13,16,29,30,31,32]. In a previous publication [16], we identified OvCa-associated HLA class I- and HLA class II-presented tumor antigens with MUC16 and MSLN representing the two main tumor antigens for HLA class I and HLA class II, respectively.

For T-cells targeting such tumor antigens, a pathophysiological relevance has already been demonstrated in several studies by correlating spontaneous, pre-existing antigen-specific T-cell responses with improved clinical outcomes [11,20,21,22]. However, the impact of tumor antigen presentation within the immunopeptidome on patient survival has not yet been investigated.

Here, we demonstrate that patients with a high presentation of MSLN in their tumor immunopeptidome, in terms of unique MSLN-derived HLA class II-presented peptides, exhibited a prolonged PFS and OS. Interestingly, we could not confirm the previously reported [33,34] negative impact of MSLN gene expression on patient survival in our cohort. This is in line with previous findings demonstrating a distorted correlation between gene expression and HLA-restricted antigen presentation, highlighting that the immunopeptidome does not mirror the transcriptome or the proteome [14,35,36,37,38]. This phenomenon has also been described for other “classical” and long-established tumor antigens, such as Her2neu, WT1, NY-ESO-1 and p53, for which only a limited or even absent HLA-bound presentation was observed, despite a tumor-associated overexpression at the mRNA level [4,5,6,7,8,9,10,11]. However, a high gene expression level of HLA-DR, which represents the main allotype for MSLN peptide presentation, as demonstrated by in silico peptide-binding predication, might be a good predictor for clinical outcomes, as we observed improved PFS and OS for respective patients. The association of a high HLA-DR expression with improved survival has already been shown for other tumor entities [39,40,41,42,43,44]. Limitations of this study include the relative low number of OvCa patients, as well as the imbalance between high-grade and low-grade tumors with 85% of the patients presented with a high-grade tumor. However, this distribution reflects the expected distribution in such a patient cohort, as approximately 80% of OvCa patients are diagnosed at an advanced stage. Nevertheless, further large cohort studies should be carried out to confirm our results [45]. Of note, the positive impact of HLA-restricted presentation of MSLN-derived peptides and HLA-DR gene expression on survival was not observed for OvCa-associated peptides in total, for MUC16-derived HLA class I-presented peptides, or the gene expression of any other HLA allele, highlighting the importance of MSLN-specific CD4^+^ T-cell-guided immune responses in OvCa. Previous studies have already demonstrated the multifaceted direct and indirect role and involvement of CD4^+^ T-cells in anti-cancer immunity [46,47,48,49,50,51,52]. These findings are in agreement with our findings and highlight the importance of the tumor antigen MSLN and the MSLN-directed CD4^+^ T-cell response in immune surveillance, specifically of OvCa. MSLN was not only described as a HLA-presented tumor antigen [16], but was also suspected to be involved in OvCa metastasis [53,54,55]. Thus, MSLN represents a potential target for novel targeted and T-cell-based therapies. Currently, MSLN-directed CAR-T-cell and antibody treatments are evaluated in early clinical studies [56,57,58] and might pave the way toward an enhanced OvCa treatment.

## 5. Conclusions

In conclusion, our study demonstrates the important role of HLA-DR peptide presentation of the tumor antigen MSLN for tumor immune surveillance and clinical outcomes of OvCa patients, and emphasizes the relevance of these peptides as targets for the development of novel immunotherapeutic approaches. Our findings further highlight the importance of immunopeptidome-guided tumor antigen discovery in general, and underscore the central role of CD4^+^ T-cell responses for anti-cancer immunity.

## Figures and Tables

**Table 1:** Characteristics of patient cohort.

	Ovarian Cancer Patients
Age on day of surgery (years)	
Range	29–93
Median	62
Menopausal status (n (%))	
Pre-/peri-menopausal	7 (18)
Post-menopausal	32 (82)
Amount of tissue employed for immunopeptidome analysis (g) Range Median	0.5–3.0 1.8
Grading (n (%)) G1/G2 G3	6 (15) 33 (85)
Nodal status (n (%)) Negative Positive n.a.	12 (34) 23 (66) 4
Distant metastases (n (%)) M0 M1	31 (79) 8 (21)
Progression-free survival (days) Range Median	38–1500 293
Overall survival (days) Range Median	38–1500 921

Abbreviations: n, number of patients; g, weight in grams; G1/2, low/intermediate tumor grading; G3, high tumor grading; n.a., not applicable; M0, no sign of distant metastases; and M1, distant metastases.

## Data Availability

The data presented in this study are available in the article and Supplementary Materials, while further details can be obtained on request from the corresponding author.

## Supplementary Materials

The following supporting information can be downloaded at: https://www.mdpi.com/article/10.3390/cancers14092260/s1, Supplementary Information, including Figure S1: Correlation between amount of tumor tissue and peptide identifications within the immunopeptidome; Figure S2: HLA presentation of tumor-associated antigenic peptides according to different clinical parameters; Figure S3: Impact of MSLN gene expression on clinical outcomes; Figure S4: Impact of HLA class I gene expression on clinical outcomes; Figure S5: Impact of HLA class II gene expression on clinical outcomes. Table S1: HLA class I- and HLA class II-restricted OvCa-associated antigens. Supplementary Data S1: In silico prediction of the 62 MSLN-derived peptides to HLA class II allotypes.
