# Supplementary material for: HLA-DR Presentation of the Tumor Antigen MSLN Associates with Clinical Outcome of Ovarian Cancer Patients
Source: Cancers (Basel). 2022 Apr 30;14(9):2260. doi: 10.3390/cancers14092260 (PMC9101593; doi:10.3390/cancers14092260)
Supplement: Supplementary file 1 [file cancers-14-02260-s001.zip › Supplementary_Information.pdf]

Supplementary Materials

for

HLA-DR presentation of the tumor antigen MSLN associates with clinical outcome of ovarian cancer patients

Christian M. Tegeler, Jonas Scheid, Hans-Georg Rammensee, Helmut R. Salih, Juliane S. Walz, Jonas S. Heitmann*, Annika Nelde

*Correspondence to: jonas.heitmann@med.uni-tuebingen.de

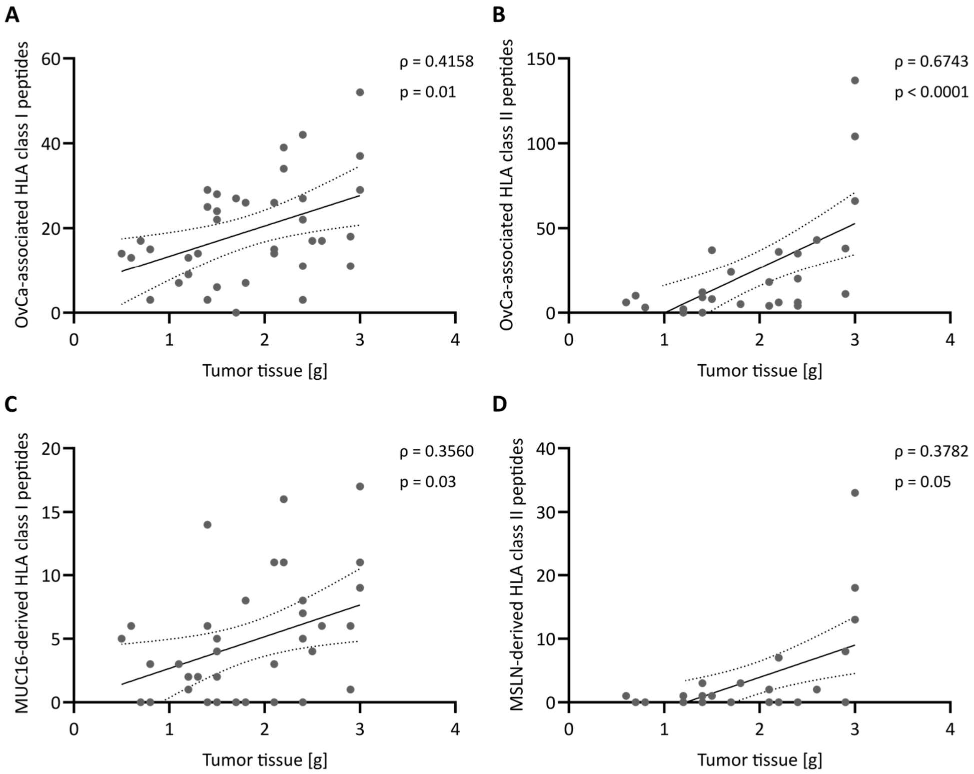

Supplementary Figure S1. Correlation between amount of tumor tissue and peptide identifications within the immunopeptidome. Correlation analysis of tumor tissue quantity used for immunopeptidome analysis to the number of identified (A) OvCa-associated and (B) MUC16-derived HLA class I-presented peptides as well as (C) OvCa-associated and (D) MSLN-derived HLA class II-presented peptides in the immunopeptidome of OvCa patients (n = 37 for HLA class I, n = 27 for HLA class II). Dots represent data from individual patients. Linear regression line with 95% confidence level (dotted lines), Spearman's rho (ρ) and p-value.

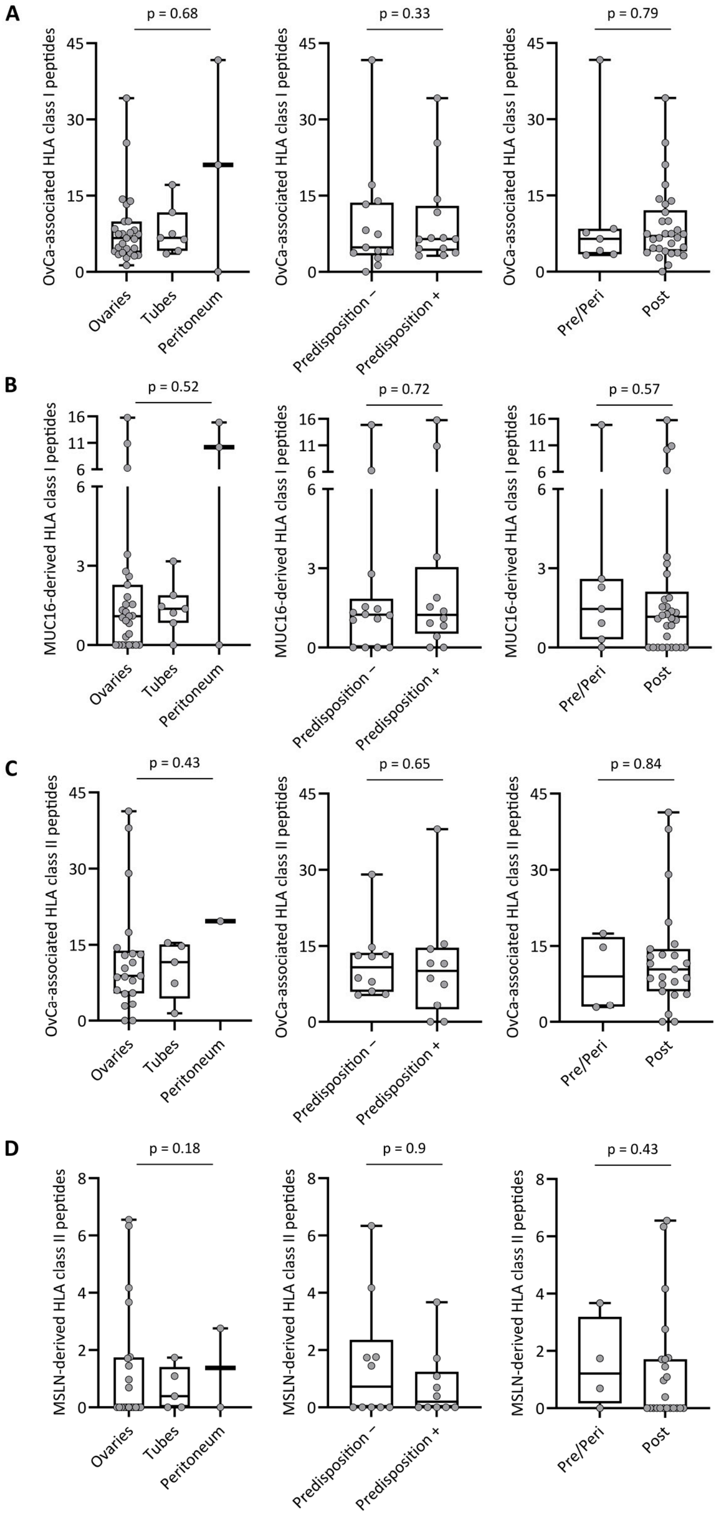

Supplementary Figure S2. HLA presentation of tumor-associated antigenic peptides according to demographics and tumor characteristics. Number of (A) OvCa-associated and (B) MUC16-derived HLA class I- as well as (C) OvCa-associated and (D) MSLN-derived HLA class II-presented peptides identified by immunopeptidome analysis of primary tumor tissue in relation to demographics and tumor characteristics,

including localization of primary tumor (left panels), menopausal status (mid panel), and familial predisposition (right panel). Dots represent data from individual patients. Boxes represent median and 25th to 75th percentiles, whiskers are minimum to maximum, Mann-Whitney U-test. Abbreviations: tubes, fallopian tubes; pre/peri, pre-menopausal/peri-menopausal; post, post-menopausal; predisposition −, no knowledge of other cancer cases in family; predisposition +, other cases of cancer in family; p, p-value.

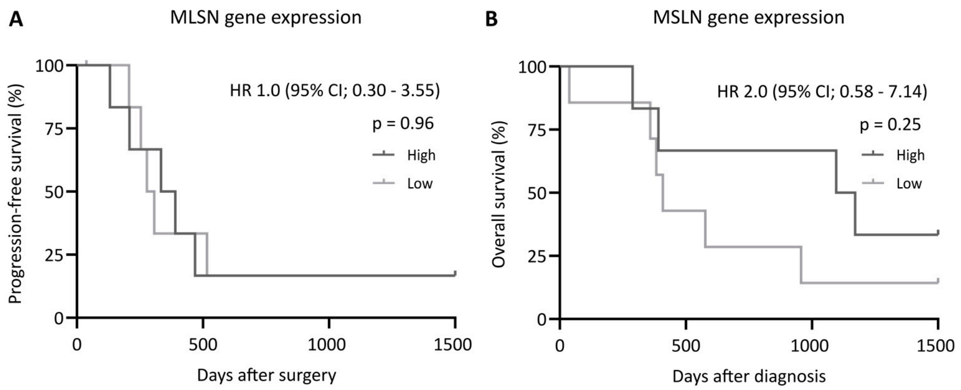

Supplementary Figure S3. Impact of MSLN gene expression on the clinical outcomes. Impact of MSLN gene expression on (A) progression-free and (B) overall survival according to MSLN gene expression in the patients' tumor. Kaplan−Meier analysis, log-rank test. Abbreviations: HR, hazard ratio; CI, confidence interval; p, p-value.

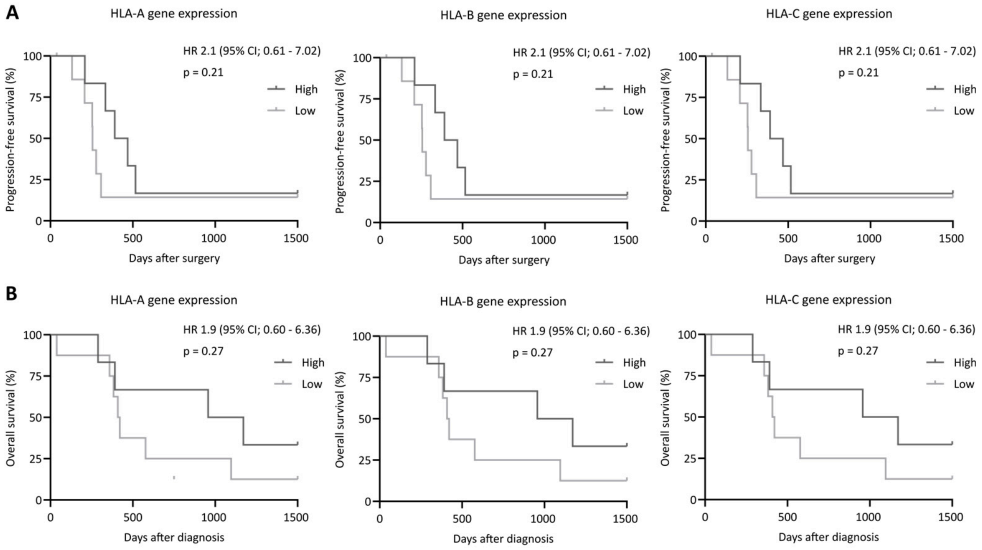

Supplementary Figure S4. Impact of HLA class I gene expression on clinical outcomes. Impact of HLA class I gene expression on (A) progression-free and (B) overall survival according to HLA class I (HLA-A, -B, and -C) gene expression in the patients' tumor. Kaplan−Meier analysis, log−rank test. Abbreviations: HR, hazard ratio; CI, confidence interval; p, p-value.

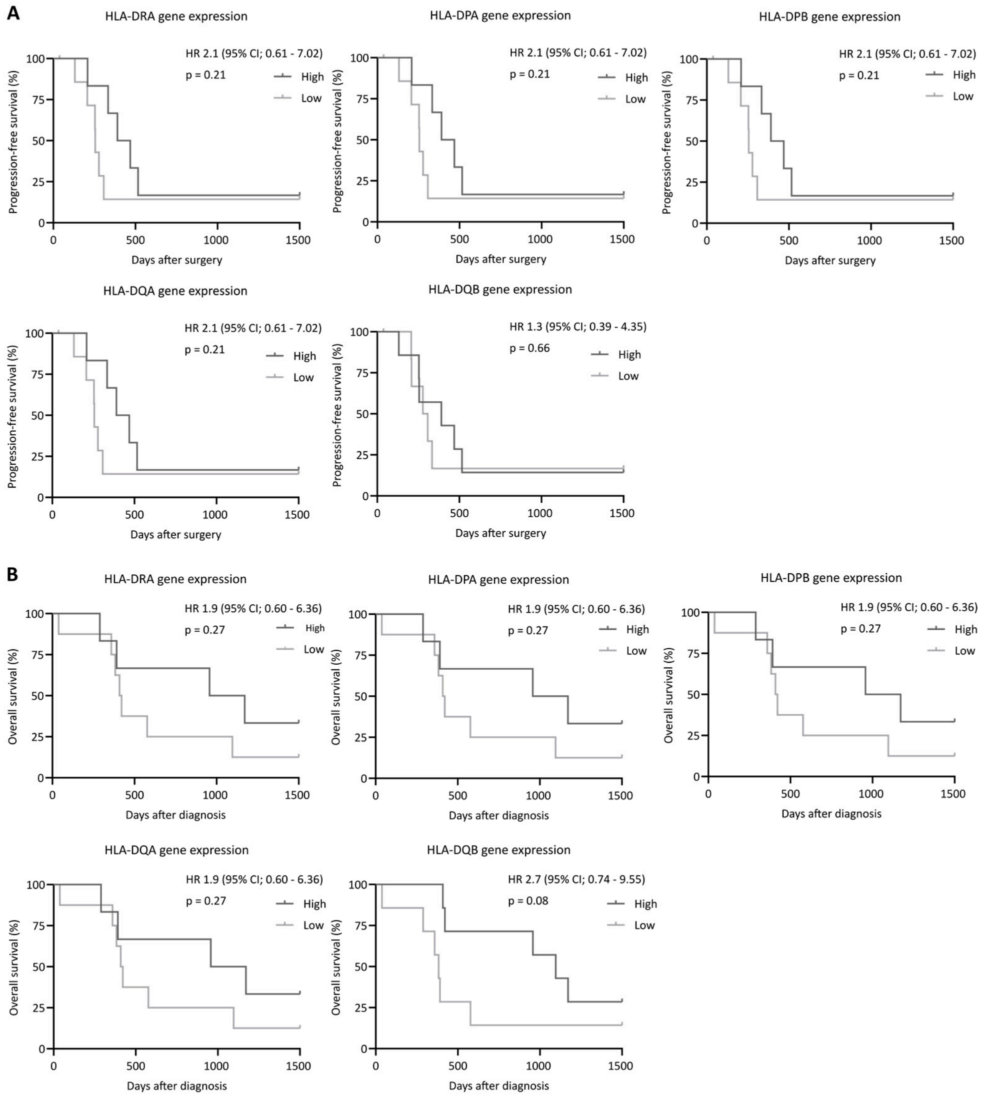

Supplementary Figure S5. Impact of HLA class II gene expression on clinical outcomes. Impact of HLA class II gene expression on (A) progression-free and (B) overall survival according to HLA class II (HLA-DRA, -DPA, -DPB, -DQA, and -DQB) gene expression in the patients' tumor. Kaplan−Meier analysis, log−rank test. Abbreviations: HR, hazard ratio; CI, confidence interval; p, p-value.

Supplementary Table S1: HLA class I- and HLA class II-restricted OvCa-associated antigens.

OvCa-associated HLA class I-restricted antigens	OvCa-associated HLA class II-restricted antigens
MUC16, EYA2, CEBPB, CRABP2, CTDSP2, CTDSL, CRABP1, S100A13, CTDSP1, AASDHPPT, LGALS1, IDO1, MMP11, TSEN34, SLC34A2, COL1A1, DNASE1, ID4, EFHC1, EPS8L1, MYOF, MANBA, ZNF217, PTTG1, PTPRF, FOLR1, FEN1, LAMC2, ATP13A5, ATP13A4, PSMG3, IFT57, BFAR, IFT172, KLK10, CXCL13, GIGYF1, DMD, P4HA1, BCAT1, UBXN1, PEG10, MFN1, SULF1, TMEM158, GOLGA1, ABHD1, SPON1, SETD8, NLRP2, DDIT4, PTTG2, PTGFRN, PPIE, FNDC3A, LRC42	MSLN, PTPRS, UBB, UBC, RL40, SQSTM, AGRG2, FPRP, PTPRF, ITB5, NGAL, FAT1, PLEC, RCN1, LAMA3, FNDC1, HTRA1, SYFB, FBLN4, PRDX2, TIG1, CXAR, FMOD, DREB, DPPA2, MUC16, NEO1, CSTN3

HLA class I- and HLA class II-restricted ovarian cancer (OvCa)-associated antigens identified previously (Schuster *et al.* PNAS 2015) to be exclusively and high frequently presented in OvCa.
